# Widespread Distribution and Evolution of Poxviral Entry-Fusion Complex Proteins in Giant Viruses

**DOI:** 10.1128/spectrum.04944-22

**Published:** 2023-03-13

**Authors:** Sheng Kao, Chi-Fei Kao, Wen Chang, Chuan Ku

**Affiliations:** a Institute of Plant and Microbial Biology, Academia Sinica, Taipei, Taiwan; b Institute of Molecular Biology, Academia Sinica, Taipei, Taiwan; Oklahoma State University

**Keywords:** *Asfarviridae*, eukaryote, *Iridoviridae*, *Nucleocytoviricota*, medusavirus, *Mimiviridae*, NCLDV, *Pithoviridae*, phylogenetics, protein evolution

## Abstract

Poxviruses are known to encode a set of proteins that form an entry-fusion complex (EFC) to mediate virus entry. However, the diversity, evolution, and origin of these EFC proteins remain poorly understood. Here, we identify the EFC protein homologs in poxviruses and other giant viruses of the phylum *Nucleocytoviricota*. The 11 EFC genes are present in almost all poxviruses, with the two smallest, G3 and O3, being absent in *Entomopoxvirinae* and basal lineages of *Chordopoxvirinae*. Five of the EFC genes are further grouped into two families, A16/G9/J5 and F9/L1, which are widely distributed across other major lineages of *Nucleocytoviricota*, including metagenome-assembled genomes, but are generally absent in viruses infecting algae or nonamoebozoan heterotrophic protists. The A16/G9/J5 and F9/L1 families cooccur, mostly as single copies, in 93% of the non-*Poxviridae* giant viruses that have at least one of them. Distribution and phylogenetic patterns suggest that both families originated in the ancestor of *Nucleocytoviricota*. In addition to the *Poxviridae* genes, homologs from each of the other *Nucleocytoviricota* families are largely clustered together, suggesting their ancient presence and vertical inheritance. Despite deep sequence divergences, we observed noticeable conservation of cysteine residues and predicted structures between EFC proteins of *Poxviridae* and other families. Overall, our study reveals widespread distribution of these EFC protein homologs beyond poxviruses, implies the existence of a conserved membrane fusion mechanism, and sheds light on host range and ancient evolution of *Nucleocytoviricota*.

**IMPORTANCE** Fusion between virus and host membranes is critical for viruses to release genetic materials and to initiate infection. Whereas most viruses use a single protein for membrane fusion, poxviruses employ a multiprotein entry-fusion complex (EFC). We report that two major families of the EFC proteins are widely distributed within the virus phylum *Nucleocytoviricota*, which includes poxviruses and other double-stranded (dsDNA) giant viruses that infect animals, amoebozoans, algae, and various microbial eukaryotes. Each of these two protein families is structurally conserved, traces its origin to the root of *Nucleocytoviricota*, was passed down to the major subclades of *Nucleocytoviricota*, and is retained in most giant viruses known to infect animals and amoebozoans. The EFC proteins therefore represent a potential mechanism for virus entry in diverse giant viruses. We hypothesize that they may have facilitated the infection of an animal/amoebozoan-like host by the last *Nucleocytoviricota* common ancestor.

## INTRODUCTION

Viral infection begins with entry into the host cell. As a prototypic member of *Poxviridae*, vaccinia mature virus binds to cell surface glycosaminoglycans and laminin (1–3) and induces actin-dependent endocytosis of virus particles (4–7). Then the endosomal low pH triggers membrane fusion between viral membrane and endosomal membrane, releasing vial core into cytoplasm (8). For enveloped viruses such as poxviruses, the timing of fusion activation is controlled by viral fusion proteins that are activated by an acidic environment. To date, most viruses use a single viral protein to trigger fusion with the host membrane (9). However, vaccinia virus (VacV) contains an entry-fusion protein complex (EFC) of 11 components, A16, A21, A28, F9, G3, G9, H2, J5, L1, L5, and O3, to execute membrane fusion during virus entry (10, 11 and references therein). Deletion of individual components of the EFC generated VacV particles with low infectivity, but how EFC mediates viral membrane fusion remains unknown (11).

*Poxviridae* belongs to the recently established phylum *Nucleocytoviricota* (12). They are nucleocytoplasmic large double-stranded DNA (dsDNA) viruses that infect all major lineages of eukaryotes, from animals and algae to amoebae and other microbes (13), and are commonly referred to as giant viruses (GVs) (14–17) for having genomes and virions comparable in size to small bacteria. GVs encode diverse proteins rarely or never found in other viruses, with very few genes widely shared across major GV lineages and most genes acquired in a lineage-specific manner at the family or lower taxonomic level (18). Virus-cell interactions, including the processes and mechanisms of cell entry, are poorly understood for most GVs. Similar to poxviruses, other GVs mostly have at least one outer envelope membrane, internal membrane, or both. In addition to animal viruses, membrane fusion has been observed at the initial stage of infection by GVs of algae (e.g., chloroviruses [19]) and other microbial eukaryotes. In amoeba-infecting GVs, infection typically begins with phagocytosis, followed by capsid opening and fusion of the internal membrane with the phagosome (17, 20). The low endosomal pH has also been suggested to induce membrane fusion for different GVs (21), including those infecting vertebrates (22, 23) and amoebozoans (24). Recent studies of African swine fever virus (ASFV) found that its pE248R (25) and pE199L (26) proteins are required for viral membrane fusion and are distant homologs of VacV EFC proteins A16/G9/J5 and F9/L1, respectively. Homologs of these proteins have also been detected in some other GVs through BLAST searches (26) and gene clustering (27), but their distribution across all the GVs, including numerous recently reported metagenome-assembled genomes (MAGs) (17, 28, 29), remains to be determined. It is also unclear whether the poxviral EFC homologs may function as an evolutionarily conserved fusion machinery in other GVs.

To better understand GV gene repertoires and virus entry, this study aims to unravel the distribution patterns, evolutionary history, and conservation of EFC proteins in poxviruses and the other GVs. Through phylogenetic analyses and sequence comparisons, our results provide insights into the origin, diversification, and duplications of EFC proteins in GVs. Furthermore, predicted models shed light on the protein structural similarity across divergent GV lineages. These findings suggest an important functional role of poxviral EFC proteins in other GVs.

## RESULTS

### Presence-absence of EFC genes.

**FIG 1:**
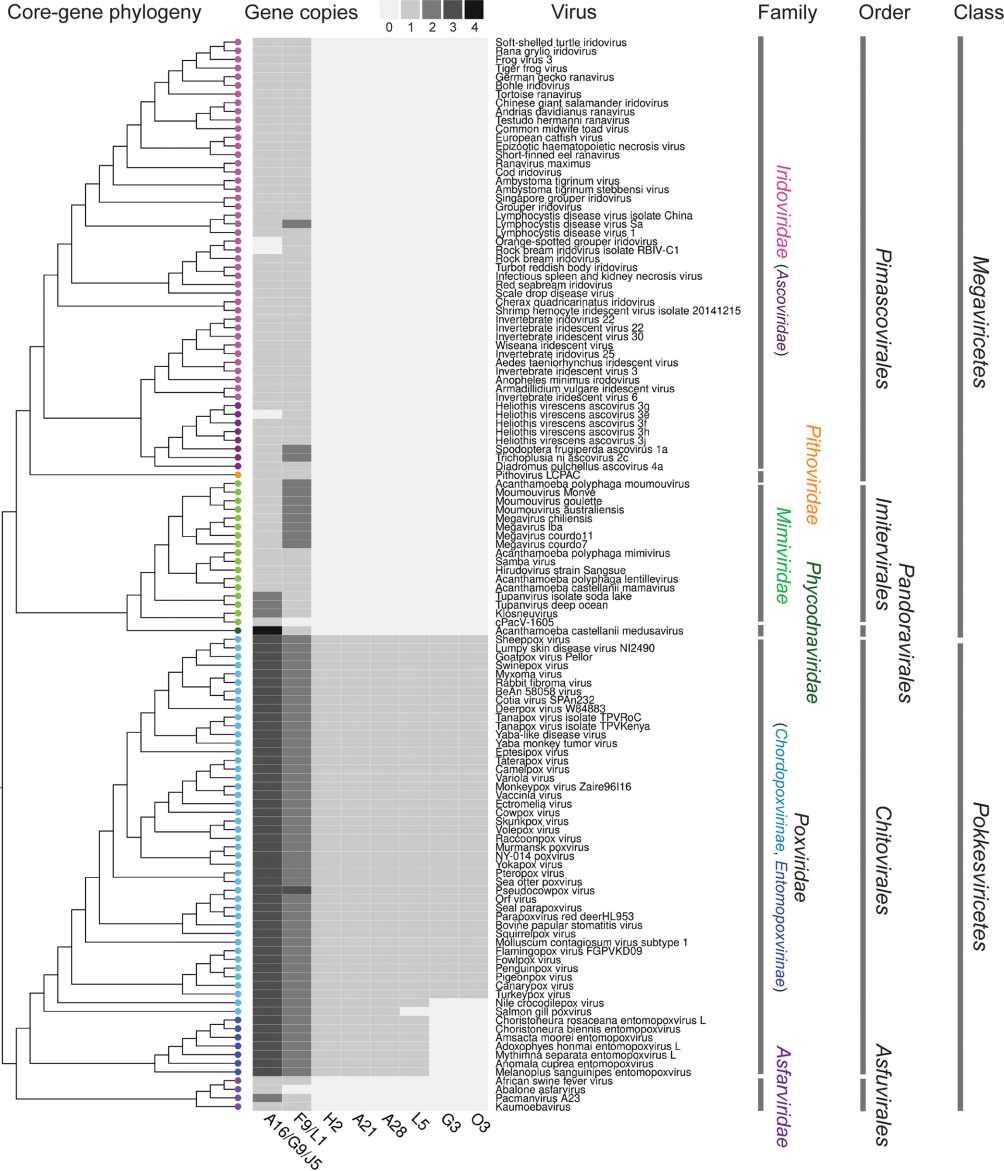
Distribution and copy numbers of entry-fusion complex (EFC) gene homologs in *Poxviridae* and other *Nucleocytoviricota* families. The 11 EFC proteins in vaccinia virus are grouped into 8 families, including one that includes A16, G9, and J5 and another that combines F9 and L1. Out of a total of 207 viruses included in a recent study (18), 124 have at least one EFC homolog, and their phylogenetic relationships based on five core proteins (18) are shown on the left. The family-level (18), order-level (30), and class-level (12) taxa are indicated on the right.

Based on a recent gene clustering data set of 207 GVs (18), mostly isolated and cultured viruses with complete or nearly complete genome sequences, we detected homologs of EFC genes in 124 GVs (Fig. 1). The 11 EFC genes are divided into 8 gene families. One gene family contains VacV A16/G9/J5 and another, F9/L1, which correspond to NCVOG1122 and NCVOG0211 (27), respectively. Most EFC genes, including A16, G9, J5, F9, L1, H2, A21, and A28, are present as a single copy in all poxviruses (except for two duplicated F9 copies in the pseudocowpox virus; Fig. 1 and Table S1 in the supplemental material). TBLASTN searches suggest that the two smallest EFC proteins, G3 and O3, are present in core *Chordopoxvirinae* but absent in salmon gill poxvirus, Nile crocodilepox virus, and *Entomopoxvirinae*. L5 is present in all poxviruses except salmon gill poxvirus. For the four additional poxviruses included in this study, we found that carp edema virus and saltwater crocodilepox virus have the same presence-absence patterns as salmon gill poxvirus and Nile crocodilepox virus, respectively. Like the avipoxviruses they are most closely related to, teiidaepox virus and cheloniid poxvirus have all the EFC genes (Table S2). Based on the core gene tree as the backbone phylogeny of GVs (Fig. 1), G3 and O3 likely originated in the common ancestor of core *Chordopoxvirinae* after its divergence from crocodilepox virus. H2, A21, A28, and L5 all trace their origins to the root of *Poxviridae*, with L5 being lost during the evolution of the salmon gill poxvirus lineage.

Homologs of A16/G9/J5 and F9/L1 are present in 124 out of the 207 GV genomes (Fig. 1). The viruses belong to both classes of *Nucleocytoviricota*, covering 6 of the 7 major family-level clades as defined previously (18) and 5 of the 6 order-level clades delineated in another study (30). Given the broad distribution across major GV lineages, it is notable that none of the isolated GVs known to infect algae have any of the EFC genes. Where A16/G9/J5 and F9/L1 genes are present, they generally cooccur, as in 93.2% (68/73) of the non-*Poxviridae* GV genomes that have at least one of them (Fig. 1). Unlike in *Poxviridae*, both gene families mostly have only one single copy in the non-*Poxviridae* genomes, with some exceptions being medusavirus (4 genes of A16/G9/J5) and moumouviruses/megaviruses (2 genes of F9/L1).

In addition to the 207 genomes of mostly isolated GVs, our search for EFC homologs extended to other GV genomes (Table S1). These include viruses in the newly proposed *Mininucleoviridae*, which have the smallest genomes (70 to 74 kb) in *Nucleocytoviricota* (31), *Marseilleviridae* genomes not included in the 207-genome data set, and various MAGs (28, 29) that have been tentatively assigned to previously proposed *Nucleocytoviricota* families or newly delineated higher-level groups called “superclades” (29). Although the MAGs tend to be more fragmented and partial genome assemblies, many of them have both A16/G9/J5 and F9/L1 genes detected in their genomes (Table S1).

### Sequence conservation and phylogenies of EFC genes.

**FIG 2:**
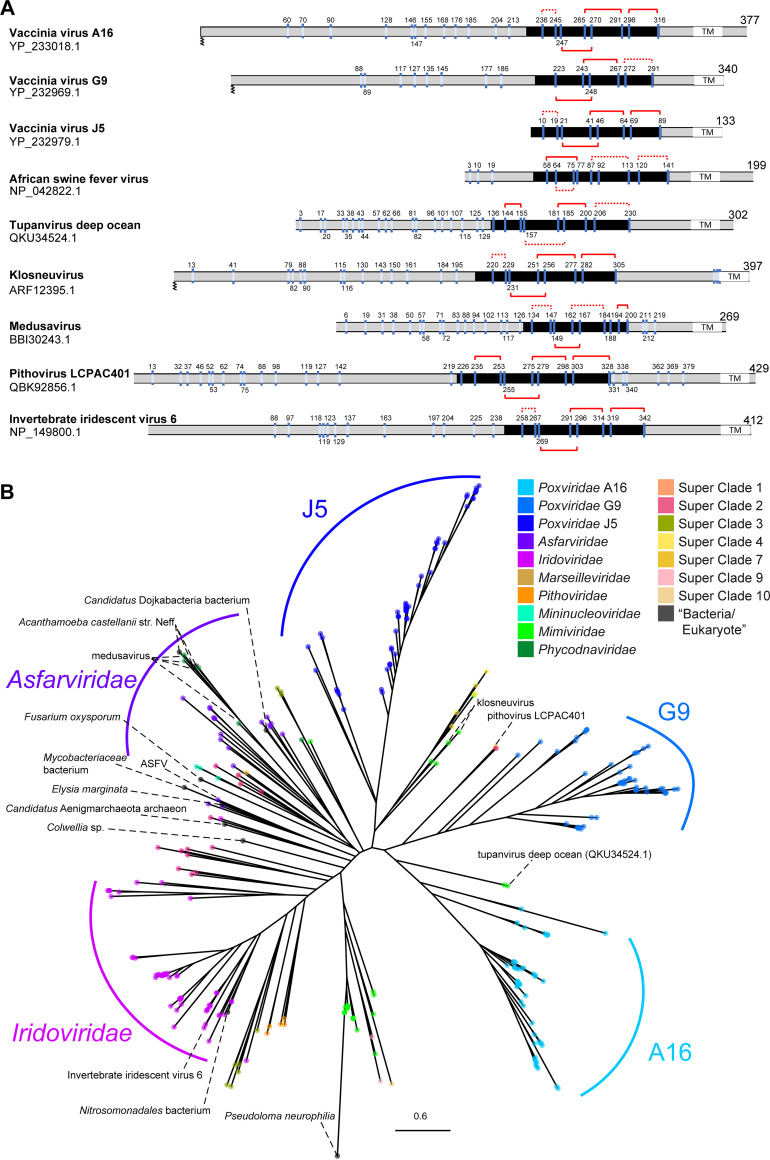
Positions of cysteine residues in representative homologs and phylogeny of the A16/G9/J5 gene family. (A) Cysteine residues are marked and numbered for representative sequences from different families. Disulfide bonds in the conserved region (black; J5-like domain) are denoted by red solid (found in structures predicted by AlphaFold2) or dotted (possible but not predicted) connections. Transmembrane domains (TM) and known or predicted N-terminal myristoylation are indicated. (B) Maximum-likelihood phylogenetic tree of A16/G9/J5 protein sequences from cultured isolates and metagenome-assembled genomes of giant viruses and the nr database of NCBI. ASFV, African swine fever virus.

**FIG 3:**
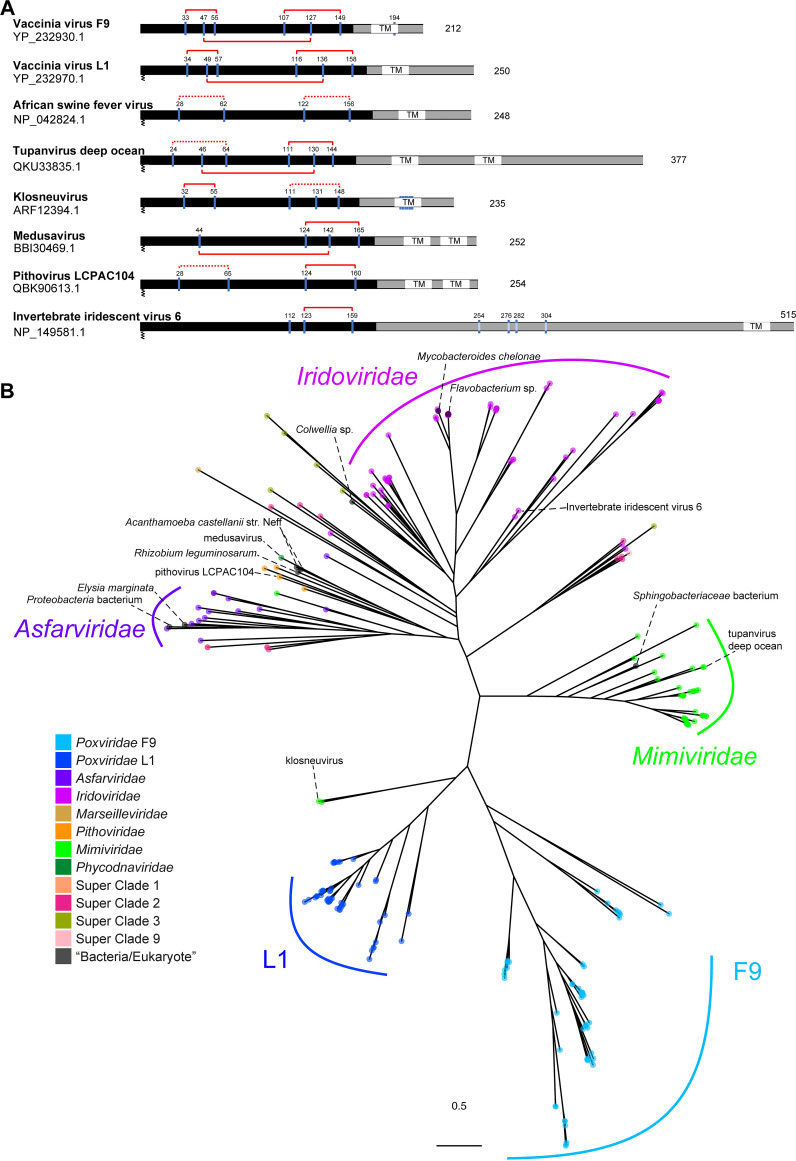
Positions of cysteine residues in representative homologs and phylogeny of the F9/L1 gene family. (A) Cysteine residues are marked and numbered for representative sequences from different families. Disulfide bonds in the conserved region (black) are denoted by red solid (found in structures predicted by AlphaFold2 or based on X-ray crystallography) or dotted (possible but not predicted) connections. Transmembrane domains (TM) and known or predicted N-terminal myristoylation are indicated. (B) Maximum-likelihood phylogenetic tree of F9/L1 protein sequences from cultured isolates and metagenome-assembled genomes of giant viruses and the nr database of NCBI.

As in poxviruses, A16/G9/J5 and F9/L1 proteins in other GVs are cysteine-rich and have predicted transmembrane domains at or near the C-terminal end (Fig. 2A and 3A). Despite sequence length variation, a conserved region is found in all A16/G9/J5 proteins, including the shortest protein, J5, in poxviruses. Similarly, a conserved region is also found in all F9/L1 proteins.

Maximum likelihood phylogenies (Fig. 2B and 3B; Data Sets S1 and S2) were inferred for both gene families from all the sequences listed in this study (Table S1). All five genes in *Poxviridae* (i.e., A16, G9, J5, F9, and L1) each form a monophyletic group, suggesting that each of these genes has a single origin, was present in the last common ancestor of *Poxviridae*, and since then has not been transferred between poxviruses and any other genomes based on the current data sets. Within both the A16/G9/J5 (Fig. 2B) and F9/L1 (Fig. 3B) trees, the closer relationships between different *Poxviridae* genes also imply that they likely share an origin to the exclusion of most non-*Poxviridae* homologs. Homologs of other GV families, including *Iridoviridae* and *Asfarviridae*, form largely monophyletic groups in both trees. *Mimiviridae* forms one major clade in both trees, except for some homologs found in klosneuviruses and tupanviruses that are more closely related to poxvirus genes. These phylogenetic patterns indicate that after the divergence of A16, G9, and J5 (Fig. 2B) or F9 and L1 (Fig. 3B) and before the last *Poxviridae* common ancestor, some transfer events took place between early poxvirus ancestors and other genomes that eventually led to the presence of these poxvirus-related homologs in some mimiviruses, superclade 4 MAGs, and pithoviruses (Fig. 2B and 3B).

A16/G9/J5 and F9/L1 homologs were also detected in non-GV genomes based on the BLASTP search against the nonredundant (nr) database (Table S1). These include Acanthamoeba castellanii strain Neff, which has been widely used as a lab host for GV isolation and cultivation. The close relationships between medusavirus and A. castellanii strain Neff in both gene family trees are consistent with the report of high gene sharing between these two genome sequences (32). The other genome sequences come from eukaryotes (Elysia marginata, Fusarium oxysporum, Pseudoloma neurophilia), one archeon (“*Candidatus* Aenigmarchaeota archaeon”), and a few bacteria. In total, these 11 A16/G9/J5 and 9 F9/L1 sequences from non-GV genomes are relatively few compared with the ones detected in GVs, and they tend to be found in nested positions within clades formed by GV sequences. Some of the non-GV genomes are incomplete assemblies, with the EFC genes found on small contigs. For example, the *Colwellia* sp. isolate is a MAG from oxic subseafloor aquifer (33) with 422 contigs (JAESPX010000000), where A16/G9/J5 and F9/L1 genes are located on a 9,732-bp contig (Table S1). Therefore, we cannot rule out that some of the EFC genes might actually be contaminating sequences from GVs which are widely distributed in various ecosystems. Given the overall patterns of distribution and phylogenetic relationships, the A16/G9/J5 and F9/L1 protein families likely originated in *Nucleocytoviricota* or were transferred to a *Nucleocytoviricota* ancestor from some other lineage that has not been sampled or has gone extinct. Since these proteins are present in major lineages of both primary branches (classes *Pokkesviricetes* and *Megaviricetes*; Fig. 1) of *Nucleocytoviricota*, their presence can be traced back to the last *Nucleocytoviricota* common ancestor (LNCA). After LNCA diversified into descendant lineages, A16/G9/J5 and F9/L1 genes duplicated in the ancestor of *Poxviridae* and have been maintained as three and two copies, respectively, throughout *Poxviridae* evolution.

### Genomic locations.

**FIG 4:**
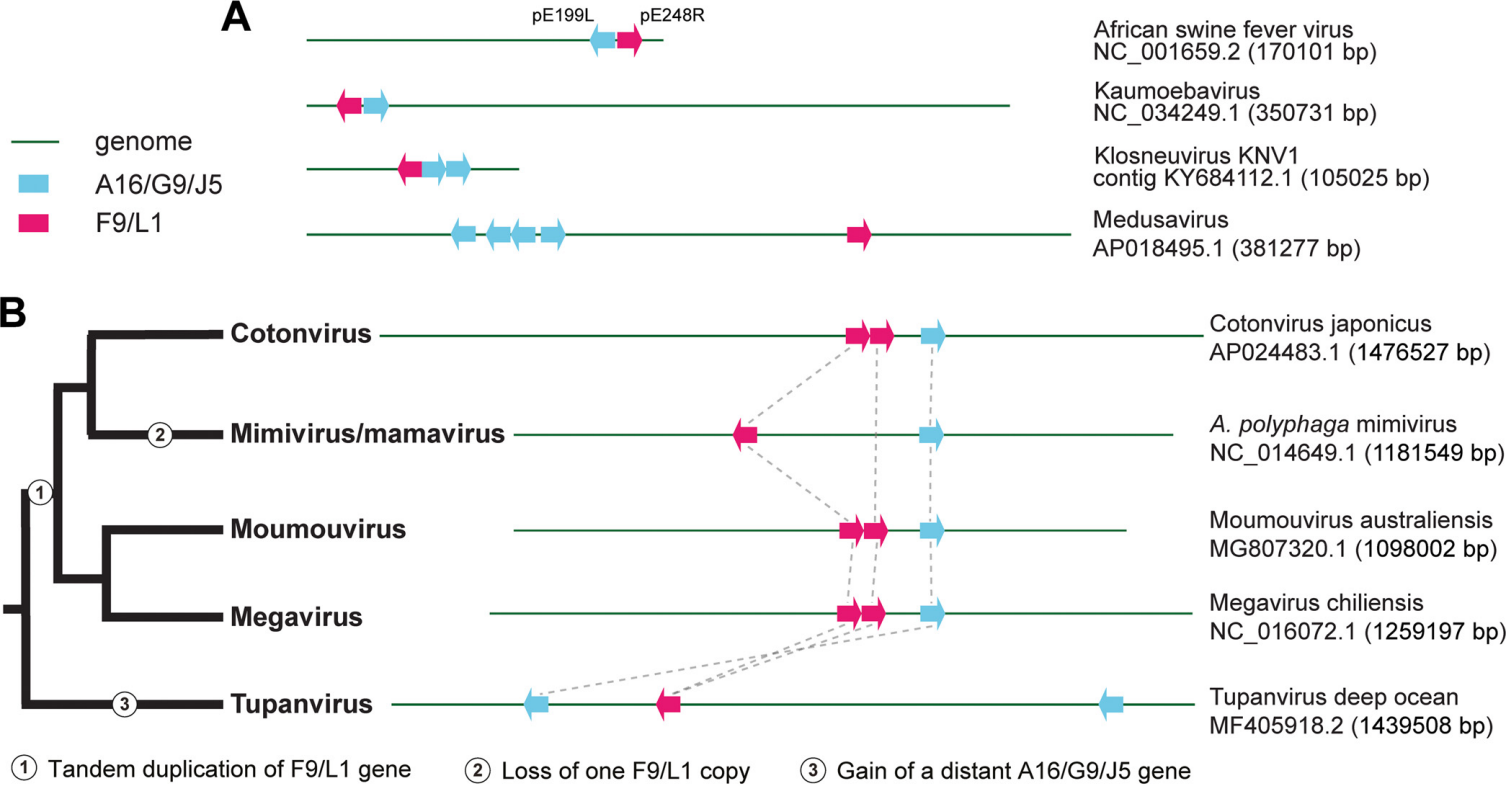
Genomic locations of A16/G9/J5 and F9/L1 genes in selected GV genomes. (A) Close proximity of both A16/G9/J5 and F9/L1 genes or multiple A16/G9/J5 genes. (B) Evolutionary dynamics of F9/L1 and A16/G9/J5 genes in a major clade of *Mimiviridae*. Dashed lines indicate orthology. Gene size and genome size are not drawn to scale.

In addition to cooccurrence of A16/G9/J5 and F9/L1 in most genomes, we observe some interesting patterns of their genomic locations (Fig. 4). A16/G9/J5 and F9/L1 genes are often in close proximity (next to each other or within a few genes), such as those in ASFV, kaumoebavirus, and klosneuvirus (Fig. 4A). The *Asfarviridae* GVs, ASFV and kaumoebavirus, both have a small open reading frame (ORF) between their A16/G9/J5 and F9/L1 genes that run in opposite directions. Similarly, in the MAG *Colwellia* sp., the A16/G9/J5 and F9/L1 copies are neighboring genes in opposite directions. Four copies of A16/G9/J5 occur within a region of 20 genes in the medusavirus genome. In a major subgroup of *Mimiviridae*, moumouviruses (lineage B), megaviruses (lineage C), and cotonvirus all have two adjacent copies of F9/L1, but there is only one copy in mimiviruses/mamaviruses (lineage A) (Fig. 4B). Based on the F9/L1 phylogeny (Data Set S2) and the phylogeny of these viruses (34), a tandem duplication of the ancestral F9/L1 gene occurred in the common ancestor of these viruses, with both copies preserved in all lineages except mimiviruses/mamaviruses, which retained only the upstream copy of the two.

### Structural conservation.

To investigate whether the identified GV EFC homologs exhibit any conserved protein structure, we took advantage of the recently reported AI system AlphaFold2 for predicting protein structure with high accuracy (35, 36). We compared the disulfide bonding patterns of non-*Poxviridae* homologs with those of VacV proteins (Fig. 2A and 3A) based on closely spaced cysteine pairs that are either known to or can potentially form disulfide bonds in the structural models. Interestingly, we found that the disulfide bonding patterns represent an evolutionarily conserved structural feature of EFC proteins.

**FIG 5:**
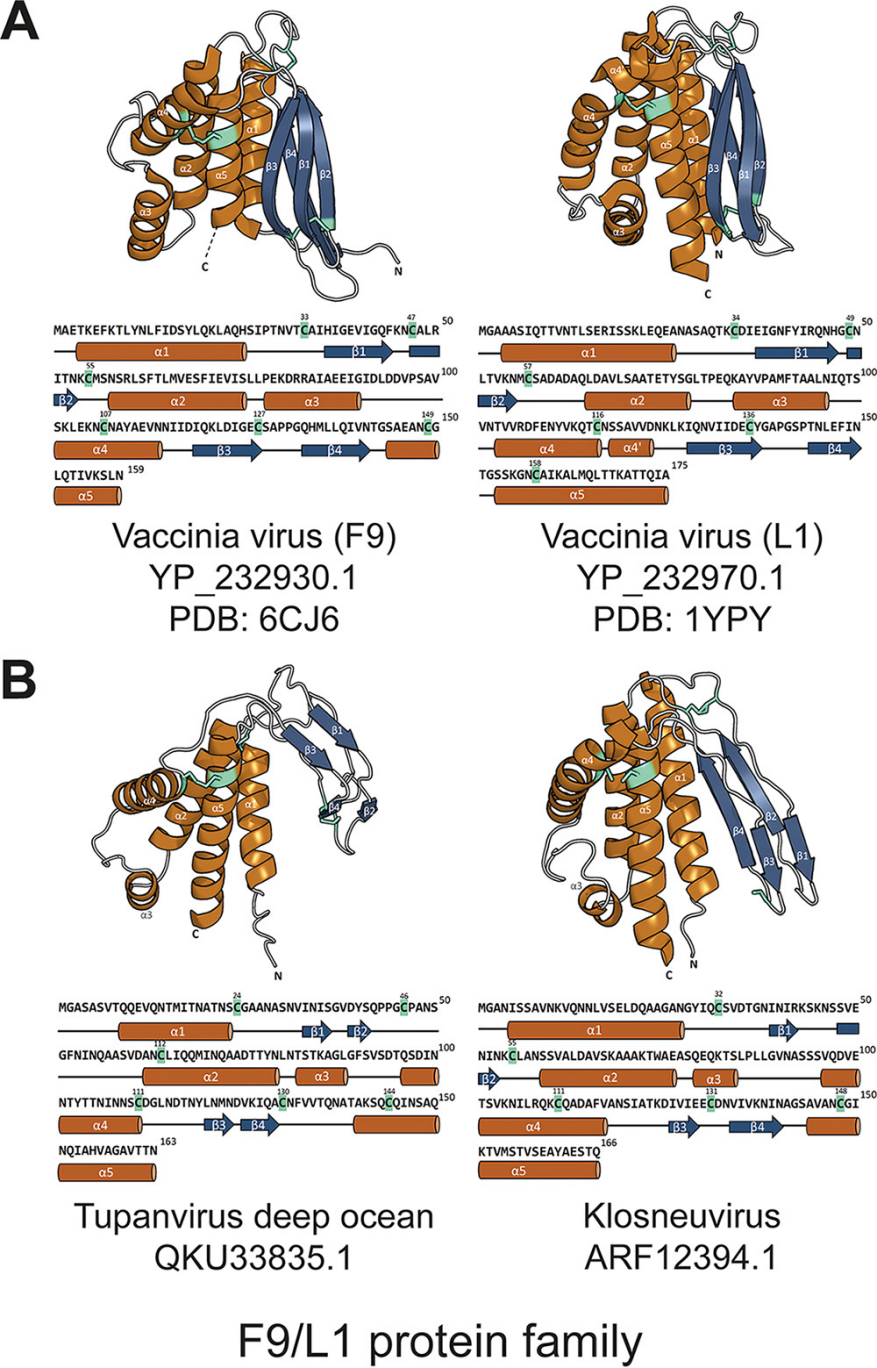
Overall structures and secondary structural elements of the N-terminal ectodomain in the F9/L1 family. (A) Cartoon representation of VacV F9 and L1 protein structures, which were determined by X-ray crystallography (37, 38). Both molecules consist of a five-helix bundle juxtaposed to two β-sheets. Three pairs of conserved disulfide bonds are found in F9 and L1. (B) Cartoon representation of AlphaFold2-predicted structures of N-terminal ectodomains of homologs in two other GVs. The modeled ectodomains, which comprise five α-helices packed against two β-sheets, exhibit structural similarity with those seen in F9 and L1. The relative positions of disulfide bonds (three pairs in QKU33835.1 and two pairs in ARF12394.1) are also similar, particularly the last pair bridging the helices α4 and α5. Cyan-green sticks indicate disulfide bonds. The α-helices and β-strands are labeled and colored orange and dark blue, respectively. The corresponding amino acid sequences annotated with secondary structural features are shown below the 3D models.

The VacV L1 was the first EFC protein with a resolved atomic structure (37) (Fig. 5). It comprises five α-helices and four β-strands (order: α1-β1-β2-α2-α3-α4-β3-β4-α5) forming a packed helical bundle juxtaposed to a pair of β-sheets (Fig. 5A). Multiple sequence alignment revealed the presence of six conserved cysteines throughout the poxviral L1 orthologs. VacV L1 has a disulfide topology of 1-3_2-5_4-6 with disulfide bonding between Cys34 and Cys57, Cys49 and Cys136, and Cys116 and Cys158 (Fig. 3A). More recently, the structure of the ectodomain of VacV F9 was also determined (38). Despite the low sequence identity (27%), superposition of the L1 and F9 ectodomains revealed a well-aligned 3-dimensional conformation with a root mean square deviation (RMSD) of 2.06 Å (see Data Set S3 at https://github.com/chuanku-lab/KaoEtAl2023_MicrobiologySpectrum/tree/main/DataS3). To validate the accuracy of AF2 prediction in EFC proteins, we also predicted the VacV L1 and F9 structures using AF2 and calculated the RMSD between the crystalized and the modeled structures upon superposition. The results showed that AF2 can accurately predict L1 and F9 structures with an RMSD of 1.75 Å across 138 Cα atoms and 2.30 Å across 143 Cα atoms, respectively.

We then used AF2 to predict the protein structures of F9/L1 homologs in two non-*Poxviridae* GVs, including tupanvirus deep ocean (QKU33835.1) and klosneuvirus (ARF12394.1) (Fig. 5B). High mean predicted local distance difference test (LDDT) scores (70.97 and 70.43, respectively) were obtained for their N-terminal ectodomains compared with the VacV L1 and F9 ectodomains. The predicted protein structure of QKU33835.1 and ARF12394.1 both contain five helices and four β-strands in the order α1-β1-β2-α2-α3-α4-β3-β4-α5, which is identical to that of VacV L1 and F9. Despite some variation in local structures, the relative positions between the helical bundle and β-sheets/loops are the same. Furthermore, although the numbers of cysteines and the corresponding disulfide bonds vary, the structural modeling suggested that the last pair of cysteines connecting the helices α4 and α5 appears to be evolutionarily conserved in the F9/L1 family.

**FIG 6:**
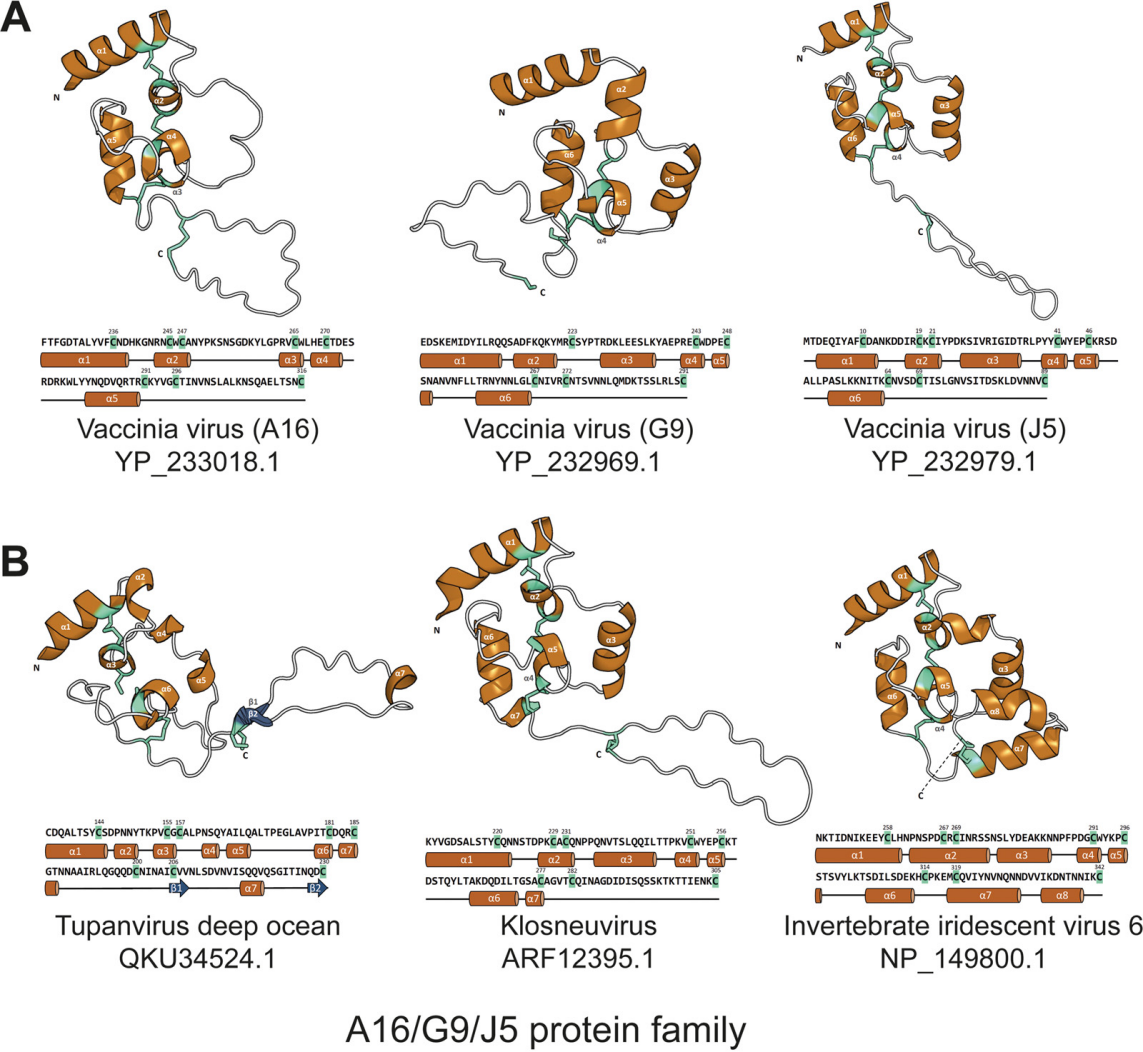
Overall structures and secondary structural elements of AlphaFold2-predicted J5-like domains in the A16/G9/J5 family. (A) VacV A16, G9, and J5. (B) Homologs from other GVs. A common fold in the J5-like domain comprises mainly α-helices and a C-terminal extended loop. There are three to four pairs of conserved disulfide bonds (cyan-green sticks) which constrain the core architecture; however, the first pair, which connects helices α1 and α2, is missing in G9. The α-helices and β-strands are labeled and colored orange and dark blue, respectively. The corresponding amino acid sequences are shown below each 3D model and annotated with the secondary structural features.

For the A16/G9/J5 family, we focused on the widely conserved structural domain (called the J5-like domain here) corresponding to the VacV J5 N-terminal ectodomain from amino acids (aa) 1 to 89 (Fig. 2A). Although none of VacV A16, G9, and J5 protein structure has been resolved, the mean pLDDT score of the J5-like domain is all above 70 (VacV A16, 75.05; VacV G9, 79.89; VacV J5, 87.01). The AF2-predicted model of the J5 ectodomain contains six short α-helices (α1 to -6) connected by loops and a long C-terminal extended loop (aa 64 to 89) (Fig. 6A). A total of eight cysteines likely form four disulfide bonds that sequentially connect the helices α1 and α2 (cys10 and cys19), helices α2 and α5 (cys21 and cys46), helices α4 and α6 (cys41 and cys64), and the C-terminal loop (cys69 and cys89) (Fig. 6A). Both the structural features and the disulfide topology of 1-2_3-5_4-6_7-8 are well conserved in the corresponding domains in vaccinia A16 and G9 protein (Fig. 6a), except that VacV G9 lacks the two cysteines forming the first pair of the disulfide bond.

We then extended the structural prediction to three other GV homologs of A16/G9/J5 (Fig. 6B). The mean pLDDT scores of the J5-like domain (Fig. 2A) in these viral homologues are all above 70 (tupanvirus deep ocean QKU34524.1, 73.13; klosneuvirus ARF12395.1, 80.67; invertebrate iridescent virus 6 NP_149800.1, 86.43), supporting that these predictions are of high confidence and accuracy. These J5-like domains in non-*Poxviridae* GVs are similarly built by short 6 to 8 α-helices that are linked by loops (Fig. 6B). Both the klosneuvirus and tupanvirus deep ocean homologs have an extended C-terminal loop. In addition, the cysteine spacing pattern also shows conserved distances between the second and third cysteines (2 residues) as well as the fourth and fifth cysteines (4 to 5 residues) that anchor the central helices and thus constrain the core region.

Besides the protein structural conservation described above, we also notice that VacV A16, G9, and L1 all have a conserved N-terminal myristoylation motif “MG” which anchor the protein in membrane and are of functional importance (39–41). The presence of the MG motif in most F9/L1 (Fig. 3) proteins implies that posttranslational lipid modification for N-terminal anchoring on membrane is well conserved in the F9/L1 family.

### Expression patterns and protein localization.

The abundant protein-coding genes in GV genomes are typically expressed in transcriptional kinetic classes that are turned on and off sequentially (42–46). Through literature research, we found that EFC genes are mostly expressed late in the transcription programs of both *Poxviridae* and non-*Poxviridae* GVs. In a transcriptomic analysis of VacV, 6 EFC genes were expressed in the late kinetic class (A16, G9, L1, A21, A28, G3), and 1 is expressed in the early/late class (F9), whereas the expression levels of 4 genes (J5, H2, L5, O3) were not well determined (45). In terms of localization, these 11 EFC proteins are all known to be embedded in the mature virion membrane of VacV (11). The ASFV A16/G9/J5 (pE199L) and F9/L1 (pE248R) proteins are both components of the virion inner envelope (26), which has been suggested to fuse with the endosomal membrane of pig cells to release the naked core containing the ASFV genome (23). While the kinetic class of pE248R remains ambivalent, pE199L is clearly a late gene (46). The insect virus Spodoptera frugiperda ascovirus 1a encodes 21 proteins that occur in the virion (47), which include its single A16/G9/J5 protein (YP_762409.1) and two F9/L1 proteins (YP_762390.1 and YP_762391.1). In mimivirus, an amoebozoan-infecting GV, its A16/G9/J5 protein (R557; YP_003987072.1) was detected in the virion proteome (48), and its F9/L1 gene (L323; YP_003986826.1) has a late gene expression promoter element (43) annotated in its genome (NC_014649.1). In the closely related sambavirus, the A16/G9/J5 protein (AMK61942.1) was also detected inside the virion (24). In medusavirus, which also infects the amoebozoan *Acanthamoeba*, the F9/L1 gene (BBI30469.1) and two A16/G9/J5 genes (BBI30243.1 and BBI30262.1) are in gene cluster 5 (the latest class), and the other two A16/G9/J5 genes (BBI30251.1 and BBI30253.1) are in cluster 4 (the second latest class) (49). To summarize, for both *Poxviridae* and other GVs that infect animals or amoebozoans, their A16/G9/J5 and F9/L1 genes tend to have late expression, and their protein products occur in the virion proteome.

## DISCUSSION

The EFC proteins A16/G9/J5 and F9/L1 have been found to be essential and nonredundant for cell entry, in particular, the membrane fusion step, by VacV (11) and ASFV (25, 26), which are the prototypical viruses of *Poxviridae* and *Asfarviridae* that comprise the class *Pokkesviricetes* of *Nucleocytoviricota*. Both the A16/G9/J5 and F9/L1 families are cysteine-rich proteins with transmembrane domains near the C-terminal end (11). In this study we show that these two generally cooccurring protein families are commonly distributed in both classes and most major lineages of *Nucleocytoviricota*. The overall conservation in the gene sequences and the predicted proteins suggests that these EFC homologs likely have a conserved function in the virus entry-fusion process across a wide range of GVs similar to that in VacV and ASFV.

One important implication for GV biology is that membrane fusion mediated by A16/G9/J5 and F9/L1 proteins is possibly a common mechanism for cell entry. Fusion with endosome or plasma membrane has been observed for many GVs infecting animal or amoebozoan cells. The absence of A16/G9/J5 and F9/L1 genes in algal GVs, from the families *Phycodnaviridae* and *Mimiviridae*, might be related to the presence of cell walls in most algae, which are an extra barrier that requires more sophisticated strategies for viral entry. For example, when the chlorovirus virion enters the *Chlorella* cell, its spike structure and enzymes first puncture the cell wall, before the virion internal membrane fuses with the cell membrane to form a tunnel for delivering viral DNA into the cytoplasm (19). However, A16/G9/J5 and F9/L1 are also absent in prasinovirus that infects wall-less green algal picoplankton (50), phaeovirus that infects wall-less gametes or spores of brown algae (51), and coccolithovirus infecting coccolithophores using an animal-like strategy (52). Although algal GVs tend to have a smaller genome size than the amoeba-infecting GVs (13), we note that the distribution of A16/G9/J5 and F9/L1 genes does not correlate with gene repertoire size, which is even smaller in poxviruses and iridoviruses that mostly have these EFC genes. Instead, a more plausible explanation for the general absence of A16/G9/J5 and F9/L1 in GVs isolated from algae is that the genes were already lost in their ancient ancestors. Based on a recent taxonomic treatment (30), the most parsimonious scenario would be complete losses of both A16/G9/J5 and F9/L1 at the origins of three lineages: (i) a subclade of *Imitervirales*, including algal viruses such as PgV, CpV (both within mesomimiviridae), TetV, and AaV, and ChoanoV infecting a choanoflagellate, (ii) *Algavirales*, including chloroviruses, prasinoviruses, and other algal viruses, and (iii) pandoravirales (or its major subclade), including coccolithoviruses, phaeoviruses, and amoeba-infecting pandoraviruses and mollivirus, where EFC genes are absent. Some smaller subclades within Mimiviridae might also have independently lost the EFC genes, such as those containing the viruses infecting the nonamoebozoan protists, *Bodo* (Discoba) and *Cafeteria* (Stramenopila). Interestingly, all GVs known to infect algae and nonamoebozoan heterotrophic microeukaryotes do not have A16/G9/J5 and F9/L1 homologs, suggesting that the loss of these entry-fusion proteins that work well in animal and amoebozoan hosts might have been the driving force for their ancestors to adapt to other hosts.

The most widely distributed genes among GVs are known to encode proteins that include DNA polymerase elongation subunit family B, D5-like primase-helicase, proliferating cell nuclear antigen, DNA-directed RNA polymerase subunits alpha and beta, poxvirus late transcription factor VLTF3, major capsid protein, and packaging ATPase (30). Although A16/G9/J5 and F9/L1 are not as commonly found in individual GVs, their distribution (Fig. 1) and phylogenetic (Fig. 2 and 3) patterns suggest their presence in LNCA. This adds to the complexity of LNCA, which, in addition to the aforementioned proteins related to DNA replication, RNA transcription, and virion assembly, possessed membrane fusion proteins for virus entry through the endocytic or plasma membrane pathway. It also implies that the host cells of LNCA were more similar to present-day cells of animals (Opisthokonta) and Amoebozoa, which both belong to one of the two major clades of eukaryotes, Amorphea (53). This agrees with the hypothesis that LNCA originated after the origin of eukaryotic cells (54) and the observation that *Nucleocytoviricota* viruses are only known to infect eukaryotes. Given the results presented in this study, A16/G9/J5 and F9/L1 are likely innovations of LNCA or its ancestors. We speculate that these genes for virus entry, along with those for DNA replication, RNA transcription, and virion assembly, could have paved the way for the descendants of LNCA to infect diverse animal (vertebrate and invertebrate) and amoebozoan (Discosea and Tubulinea) hosts and set the stage for later genome gigantism.

To date, mechanisms of viral membrane fusion have only been proposed for the fusion process induced by enveloped viruses carrying only one fusion protein (9). How poxviruses use an 11-protein complex to mediate membrane fusion remains a mystery. Nevertheless, the basic principles of membrane fusion are likely similar, such as the presence of a hydrophobic fusion loop or fusion peptide for insertion into host membrane and the conformational change that breaks the energy barrier and ultimately merges two membranes. Accordingly, if A16/G9/J5 and F9/L1 were the components in ancestors of GVs that worked together to drive membrane fusion, either one of them should contain the fusion loop or fusion peptide. Foo et al. (55) proposed a myristoyl switch model for VacV L1 in which the buried N-terminal myristate moiety in the L1 helix α1 swings up upon activation and the exposed N-terminal lipid group acts as an anchor to connect the host membrane. Interestingly, although both poxviral A16 and G9 harbor an N-myristoylation motif, MG, this lipid modification seems more conserved in the F9/L1 family of GVs (Fig. 2 and 3). In addition, a recent study showed that abrogation of VacV L1 N-myristoylation is more detrimental to the virus than inhibiting the same modification on A16 or G9 (40). Further studies of L1 are warranted to unveil the function of L1 N-myristoylation and its role in GV entry. Also of note, the presence of A16/G9/J5 homologs in many GVs raises the question of whether proteins similar to the VacV fusion suppressers, namely, A26 (56) and A56/K2 complex (57), which interact with VacV A16/G9 subcomplex to regulate fusion, also exist in other GVs.

In this study, we aimed to extend a homology search from sequence to structural conservation. We therefore employed AlphaFold2 to predict 3-dimensional structures of selected EFC protein homologs and compared structural similarity by superposition. Strikingly, after selecting models with high pLDDT scores, we found remarkable structural homology in both A16/G9/J5 and F9/L1 families between VacV and distantly related GVs, such as klosneuvirus and tupanvirus deep ocean (Fig. 5 and 6). As mentioned above, both the A16/G9/J5 and F9/L1 families comprise cysteine-rich proteins, and the putative disulfide bonding patterns appear conserved across GVs (Fig. 2 and 3). Therefore, we deduced that the intramolecular disulfide bonding may be associated with an evolutionarily conserved entry-fusion mechanism for GV entry.

In addition to implications for protein conservation, host range, and ancient evolution, this study highlights A16/G9/J5 and F9/L1 proteins in GVs as targets for further biochemical and molecular biological characterization. Unlike other GVs, poxviruses are highly conserved in having three A16/G9/J5 and two F9/L1 genes (Fig. 1). In addition to individual protein structures (Fig. 5 and 6), their structural and functional roles in the entire poxvirus EFC remain to be elucidated. The general cooccurrence of A16/G9/J5 and F9/L1 genes in other GVs (Fig. 1) also suggests that their protein products might interact and coevolve with each other. Some intriguing examples of the close proximity of A16/G9/J5 and F9/L1 genes and their conserved genomic localizations (Fig. 4) merit further investigation of their roles in expression and functional regulation. With the importance of GVs for public health, agriculture, wildlife populations, and environmental microbes, a comprehensive view of their entry-fusion proteins is greatly needed and pivotal for understanding their biology and evolution.

## MATERIALS AND METHODS

### Identification of EFC homologs.

To identify genes across giant viruses that encode homologs of EFC proteins, we searched for orthogroups (gene clusters) containing individual VacV EFC genes in the all-against-all gene clustering data set of a recent study of gene contents of 207 GV genomes (18), including 51 from *Poxviridae*. In addition to these genomes, we added 4 genomes covering underrepresented lineages of Poxviridae, including carp edema virus strain FTI2020 (58), saltwater crocodilepox virus subtype 1 (59), teiidaepox virus 1 (60), and cheloniid poxvirus 1 (61). The presence-absence patterns of the EFC genes in all poxviruses were verified through a literature search and using TBLASTN (BLAST v2.6.0 [62]) with VacV EFC proteins against the other poxvirus genome nucleotide sequences. For nonpoxvirus genomes, EFC gene homologs in *Asfarviridae*, *Iridoviridae*, *Mimiviridae*, and *Pithoviridae* reported previously (26, 63) were included and used to find other EFC homologs that were clustered together in the same orthogroups. Furthermore, we used these EFC homologs encoded by the predominantly cultured viruses as queries and performed a BLASTP (62) search against predicted proteins in two data sets of MAGs of environmental giant viruses sampled from diverse ecosystems (28, 29) (query coverage, ≥30%; e value, ≤1 × 10^−5^). A similar BLASTP search (query coverage, ≥30%; alignment length, ≥100; e value, ≤1 × 10^−5^) against the NCBI nonredundant protein database (64) was used to detect any additional EFC homologs in genome sequences of other giant viruses, other viruses, and cellular organisms (excluding hits to highly repetitive sequences or parts absent in poxvirus homologs).

### Phylogenetic analyses.

The identified sequences in each of the two protein families, A16/G9/J5 and F9/L1 (Table S1), were aligned using MAFFT v7.310 (65) with the default parameters. Maximum likelihood trees were constructed from the alignments using IQ-TREE v2.2.0 (66) with the best-fit model selected using ModelFinder (67). The trees were visualized using Figtree v1.4.4 and the package ggtree v3.4.4 (68) in RStudio (2022.02.3 build 492).

### Structural modeling.

The VacV F9 (PDB: 6CJ6) (38) and L1 (PDB: 1YPY) (37) protein structures were previously determined by X-ray crystallography. We predicted the structure of the F9/L1 proteins of selected GVs using AlphaFold2 (AF2) web server v1.4 (https://colab.research.google.com/github/sokrypton/ColabFold/blob/main/AlphaFold2.ipynb; accessed October 2022) with the default settings without AMBER relaxation or PDB templates (35, 36). All sequence alignments and templates were obtained using MMseqs2 (69). In order to evaluate the model quality, the predicted local distance difference test (pLDDT) score of each amino acid (aa) was calculated. Only models having both the mean pLDDT score and half of the residue pLDDT scores above 70 were retained for further analyses. The highest-scored model of each F9/L1 protein was superimposed with the VacV L1 protein structure using PyMOL Molecular Graphics System v1.8 (70). Protein structure predictions of A16/G9/J5 homologs were also performed using AF2 with the same parameters and criteria. Because of the high sequence divergence and length variation, our structural comparisons focused on the conserved N-terminal ectodomains of F9/L1 (VacV L1 aa 1 to 175) and A16/G9/J5 (VacV J5 aa 1 to 89), which contain characteristic disulfide bonds suitable for protein superposition comparison. The root mean square deviation (RMSD) value was calculated in PDBeFold using the secondary-structure matching (SSM) tool (71). All the protein structural and modeling drawings were prepared using PyMOL as described in the user manual.
